# Safety Net Provider Attitudes Toward Smoking Cessation Treatment

**DOI:** 10.3389/fpsyt.2021.744816

**Published:** 2021-09-28

**Authors:** Lindsay R. Meredith, Wave-Ananda Baskerville, Theodore C. Friedman, Brian Hurley, Tasha Dixon, Norma Mtume, Luz Rodriguez, Briana Lopez, Susan Hsieh, Lara A. Ray

**Affiliations:** ^1^Department of Psychology, University of California, Los Angeles, Los Angeles, CA, United States; ^2^Department of Internal Medicine, Charles R. Drew University, Los Angeles, CA, United States; ^3^Los Angeles County Department of Health Services, Los Angeles, CA, United States; ^4^Friends Research Institute, Cerritos, CA, United States; ^5^Brain Research Institute, University of California, Los Angeles, Los Angeles, CA, United States; ^6^Department of Psychiatry and Biobehavioral Sciences, University of California, Los Angeles, Los Angeles, CA, United States

**Keywords:** tobacco use, smoking cessation, nicotine, provider attitudes, public health agency

## Abstract

**Background:** Cigarette smoking, which poses significant health risks, is prevalent among vulnerable populations commonly treated by safety net providers. A large-scale implementation science project on specialty tobacco use treatment was launched within the Los Angeles County Health Agency. The first phase of this study seeks to summarize and compare smoking cessation treatment attitudes of providers at the Department of Health Services (DHS) and Department of Mental Health (DMH).

**Methods:** In total, 467 safety net health care providers (DHS = 322; DMH = 145) completed a survey inquiring about attitudes on smoking cessation treatment consisting of locally developed items and those informed by a scale on readiness for organizational change. Descriptive statistics and non-parametric tests were conducted to examine treatment attitudes for DHS and DMH providers.

**Results:** Between agencies, providers largely reported similar attitudes on smoking cessation treatment and expressed positive beliefs regarding the efficacy of smoking cessation aids. Providers slightly or moderately agreed with being prepared to identify and diagnose tobacco use among patients. DMH providers stated that identification of tobacco use was less in line with their job responsibilities (*p* < 0.0001) and less strongly agreed that varenicline is effective for smoking cessation (*p* = 0.003), compared with DHS providers.

**Conclusions:** Providers supported smoking cessation aid efficacy but may benefit from additional training on identification and treatment of tobacco use. These findings support the implementation of specialty tobacco cessation treatment programs with training on medications in safety net health care systems, which has the potential to yield large-scale public health benefits.

## Introduction

Publicly-administered health agencies play a large role in the delivery of health services, including smoking cessation treatment, to populations who receive safety net medical care. The Los Angeles County Health Agency is the second largest municipal health system in the nation, serving a diverse group of 750,000 patients annually (1). It consists of three service-providing entities, the Department of Health Services (DHS), the Department of Mental Health (DMH), and the Department of Public Health (DPH). All three entities treat Medi-Cal beneficiaries and other low-income patients, with DHS focusing on physical health and treating over 500,000 patients annually, DMH focusing on mental health and treating over 250,000 patients annually, and DPH focusing on population health. Although distinct, agency providers and representatives from all three service-providing entities communicate and work together (1).

California has a long history of smoke-free regulations, being the first state to prohibit smoking in the workplace (2). Despite this history, between the 1990s and 2010s, a moderate increase in light and intermittent smoking (i.e., consuming 1–5 cigarettes per day) was observed in California (3). While California's regulations and efforts to decrease tobacco use have been successful in the general population, cigarette smoking remains elevated among certain groups. For instance, rural residents of California are more likely to smoke tobacco and have lower cessation rates than those residing in urban areas (4, 5). Moreover, individuals at the greatest risk for developing a TUD in California are among those experiencing high levels of stress and racial discrimination, males, certain racial and ethnic groups, and those with lower education, income, employment, and rates of health insurance (4). This is consistent with a nationwide pattern, whereby cigarette smoking, due to a multitude of factors, is more prevalent among individuals with lower education, lower socioeconomic status, and mental illness (6–9). These characteristics are reflective of patients served by the Los Angeles County Health Agency, who are disproportionately affected by tobacco use disorder (TUD).

Nicotine is highly addictive, and cigarette use contributes to over 400,000 premature deaths in the United States annually (10, 11). Individuals who use tobacco and have lower socioeconomic status and mental illness are shown to experience the worst health outcomes (6). Germane to TUD, negative health consequences of tobacco use extend to both mental and physical health. For instance, individuals with mental health disorders have less success in reaching smoking cessation (8). Additionally, persons with mental health conditions are more vulnerable to continued and heavy tobacco use, as nicotine use is commonly used to “self-medicate” in attempts to assuage symptoms of mental illness (8, 12). Regarding the negative health effects of cigarette smoking, ~40% of those who smoke will die prematurely (11). Common causes of premature deaths by smoking or secondhand smoke exposure are cardiovascular diseases, metabolic diseases, pulmonary diseases, and lung cancers. It is evident that the opportunity and need to address patient's tobacco use will arise in both mental and physical health care settings, especially those serving populations at the highest risk. Further, the prevalence and rate of negative health outcomes of cigarette smoking is higher among individuals with public health insurance, such as those accessing Los Angeles County Health Agency services with Medi-Cal (6).

In light of the findings reviewed above, the Los Angeles County Health Agency aims to take active steps to better reduce tobacco-related mortality and to confront health disparities among these vulnerable populations. Approaches to address these concerns include assessing provider attitudes toward smoking cessation treatment and testing and improving intervention efforts. Research suggests that primary care provider attitudes are important indicators of success, such that beliefs surrounding smoking cessation counseling are positively associated with their own counseling and referral behaviors in addition to increased patient-reported quit rates (13). Further, direct provider advice and referral is vital to higher smoking cessation rates (14). A meta-analytic report shows that mental health staff specifically express low confidence in addressing smoking cessation among individuals with mental illnesses and this work highlights the need for routine provider training and promotion of harm reduction strategies (12). In return, characterizing physical health and mental health care provider attitudes toward tobacco use identification, diagnosis, and treatment may be a necessary first step toward improving care in safety net settings and to discern agency strengths and areas for improvement. Health professionals are at the forefront of the tobacco epidemic (15). Los Angeles County Health Agency patients routinely consult with both DHS and DMH providers about their tobacco use and these interactions may function as one of the limited health service opportunities for TUD to be addressed (1).

In order to improve smoking cessation services for individuals treated by the Los Angeles County Health Agency, a large-scale dissemination and implementation science project was launched in which clinics within DHS and DMH were randomly assigned to receive specialty tobacco use disorder services (STUDS) or treatment as usual (TAU). While this project is currently underway, the first phase of the study, which consists of evaluating baseline attitudes and beliefs of providers about smoking cessation diagnosis and treatment, including medications, is complete. Therefore, the present study sought to summarize and compare smoking cessation treatment attitudes and beliefs of providers from DHS and DMH clinics. This comparison offers unique insight into differences between physical health and mental health care providers' attitudes toward smoking cessation within the same public health agency. Given their prescriber role, we hypothesized that DHS providers would believe various smoking cessation aids to be more effective than DMH providers. In line with previous research, we hypothesized that DMH providers would less strongly agree that they are prepared to identify, diagnosis, and treat individuals with TUD. Lastly, we hypothesized that the majority of DMH and DHS providers would express interest in receiving additional training on tobacco cessation treatment.

## Methods

### Sample and Procedures

In total, 467 staff members (322 members from DHS and 145 members from DMH), in which STUDS or TAU could be administered, voluntarily completed the survey without compensation. Surveys were administered on paper to all 757 staff members within six DMH clinics and six DHS clinics. Survey responses were later transcribed into Qualtrics, an online survey platform. The response rate was 62% overall, where 49% of providers within DMH clinics and 70% of providers within DHS clinics completed the survey. Both departments are safety net clinics for a major portion of Medi-Cal beneficiaries in Los Angeles County and are outpatient facilities. Typical provider roles at DHS clinics include primary care physicians, primary care nurse practitioners, behavioral health specialists, and nursing staff, along with counselors, social workers, and community health workers. Typical provider roles at DMH clinics include psychiatrists, psychologists, counselors, social workers, and community health workers.

### Measures

The survey was developed using both locally-developed items specific to this research project, as well as items informed by a scale on readiness for organizational change (16). Beliefs and attitudes about smoking cessation treatment were measured on a 5- and 7-point Likert scale, respectively, as seen in Tables 1, 2. Regarding attitudes, respondents were asked to report how much they agreed with items relating to smoking cessation treatment from “strongly disagree” (1) to “strongly agree” (7) with “neutral” (4) falling in the middle. In terms of beliefs, respondents reported on how effective they believed certain smoking cessation treatments to be from “not at all effective” (1) to “very effective” (5) with “neutral” (3) falling in the middle. Notably, pharmacotherapies included in these belief items have demonstrated efficacy as smoking cessation aids through clinical trials (17). In addition to the Likert scale, a response option of “do not know” was available for respondents to select but these “do not know” responses were excluded from statistical analyses in order to utilize an ordinal dataset. Discrepancies in the number of individual survey item responses are attributed to missing responses and excluded “do not know” responses.

**Table 1:** Comparing smoking cessation treatment attitudes between DHS and DMH health agency providers.

**Specific survey item**	**Responses (*N*)**	**Overall (*****N*** **=** **467)**		**DHS (*****n*** **=** **322)**		**DMH (*****n*** **=** **145)**		***p*-value**
		**Median**	**% “Agree”**	**Median**	**% “Agree”**	**Median**	**% “Agree”**	
**Attitudes about Smoking Cessation Treatment**								
**(Rated on 1–7 Likert scale: 1** **=** **Strongly Disagree, 4** **=** **Neutral, 7** **=** **Strongly Agree)**								
Identifying patients who smoke tobacco fits my job description.	440	6	66.6%	6	71.2%	5	56.5%	**0.00003**
Treating/supporting the treatment of smokers with medications fits my job description.	430	6	62.8%	6	64.5%	5	59.3%	0.075
I find it difficult to discuss my patients' use of tobacco with them.	420	2	16.7%	3	20.0%	2	9.7%	0.056
I am prepared to identify or diagnose my patients who smoke tobacco.	410	6	65.1%	6	64.4%	5	66.7%	0.255
I am prepared to discuss my patients' use of tobacco with them.	415	6	69.6%	6	67.0%	6	75.2%	0.178
Medications for smoking cessation can be effective without behavioral health counseling.	392	3	21.7%	4	23.5%	3	18.0%	0.080

*DHS, Department of Health Services; DMH, Department of Mental Health; bold p-value denotes significantly different response distributions (Bonferroni corrected at p **<** 0.0045) between DHS and DMH agencies according to Wilcoxon Rank Sum Test; % “Agree” indicates percentage of providers responding with “5,” “6,” or “7”; N differs for each survey item depending on response rates*.

**Table 2:** Comparing smoking cessation aid beliefs between DHS and DMH health agency providers.

**Specific survey item**	**Responses (*N*)**	**Overall (*****N*** **=** **467)**		**DHS (*****n*** **=** **322)**		**DMH (*****n*** **=** **145)**		***p*-value**
		**Median**	**% “Effective”**	**Median**	**% “Effective”**	**Median**	**% “Effective”**	
**Beliefs about the Efficacy of Smoking Cessation Aids**								
**(Rated on 1–5 Likert scale: 1** **=** **Not at all Effective, 3** **=** **Neutral, 5** **=** **Very Effective)**								
In your opinion, how effective is …								
Nicotine patch?	304	4	64.1%	4	64.1%	4	64.2%	0.428
Nicotine gum?	289	4	56.1%	4	55.1%	4	58.2%	0.927
Nicotine lozenges?	271	4	53.5%	4	51.6%	4	57.7%	0.890
Varenicline?	271	4	68.3%	4	73.7%	4	54.6%	**0.003**
Bupropion?	254	4	67.7%	4	70.2%	4	61.8%	0.191

*DHS, Department of Health Services; DMH, Department of Mental Health; bold p-value denotes significantly different response distributions (Bonferroni-corrected at p **<** 0.0045) between DHS and DMH agencies according to Wilcoxon Rank Sum Test; % “Effective” indicates percentage of providers responding with “4” or “5”; N differs for each survey item depending on response rates*.

### Analyses

Descriptive statistics characterize the degree to which providers across agencies identified or agreed with various attitudes and beliefs regarding smoking cessation treatment and are provided in the corresponding tables and figures. Statistical analyses comparing the full range of attitude and belief Likert responses between the two agencies were conducted using Wilcoxon Rank Sum Test, a non-parametric method comparing two independent groups that is suitable for non-normally distributed ordinal data (18). For each survey item presented in Tables 1, 2, analyses tested whether response distributions significantly differed between providers at DHS vs. DMH clinics and include the two-sided asymptotic *p*-value corresponding to the Wilcoxon Rank Sum statistic. The Hodges-Lehmann estimate for the difference of the location parameter and corresponding asymptotic 95% confidence interval were calculated and are provided for significant items (19). In addition, a second set of analyses were conducted using Chi-square Test of Independence, which is a non-parametric test appropriate for nominal data (20), to compare the proportion of providers from DHS vs. DMH clinics who responded in agreement to each attitude and belief item. Specifically, for attitude items, responses were coded and dichotomized into “disagree” (0) and “agree” (1), such that raw responses ranging from “strongly disagree” (1) to “neutral” (4) were coded as “disagree,” and raw responses ranging from “slightly agree” (5) to “strongly agree” (7) were coded as “agree.” For belief items, responses were coded and dichotomized into “not effective” (0) and “effective” (1), such that raw responses ranging from “not at all effective” (1) to “neutral” (3) were coded as “not effective,” and raw responses ranging from “moderately effective” (4) to “very effective” (5) were coded as “effective.” Chi-square test statistics and *p*-values were calculated and are provided for significant items. To reduce type 1 error rate for multiple comparisons, a Bonferroni family-wise correction for each set of analyses was set at *p* < 0.0045, where α′ = α/c, # of comparisons (c) = 11. Descriptive and Chi-square analyses were completed in SAS Statistical Software [Version 9.4] (21) and Wilcoxon Rank Sum statistics were completed in R Studio, [Version 1.4.1103] using the “exactRankTests” package.

## Results

Across all clinics, 41% of providers reported practicing in their field for >10 years, 36% reported practicing 2–10 years, and 22% reported practicing <2 years (*n* = 380 respondents), with DMH clinics tending to have more providers with >10 years of experience than DHS clinics. A proportion of survey respondents self-reported their job roles, which match the expected roles reported above (see Sample and Procedures), and additionally includes other specified roles such as clinical pharmacist, administrative clerk, medical interns, substance abuse counselor, and recreation therapist. Median survey item responses and agreement percentages for provider smoking cessation attitudes and beliefs across the DHS and DMH agencies are presented in Tables 1, 2. Additionally, both tables provide the statistical significance of each comparison by agency, DHS vs. DMH, for the Wilcoxon Rank Sum test. In terms of attitudes, overall responses across agencies, show that providers “moderately agree” (i.e., median score of 6 points on a 7-point Likert scale) with statements about being prepared to discuss, identify, or diagnose patients' tobacco use (65–69% of providers agreed). According to the Wilcoxon test, providers in the DHS agency reported significantly higher agreement ratings with the statement, “identifying patients who smoke tobacco fits my job description,” W = 25,864, *p* = 0.00003; Hodges-Lehmann difference estimate 1.00 [95% CI: 0.00009, 1.00003]. Similarly, a significantly greater proportion of DHS providers (71.2%) than DMH providers (56.5%) agreed with this statement on job role, χ^2^ (1, *N* = 440) = 9.16, *p* = 0.0025. While a smaller proportion of DMH providers (9.7%) vs. DHS providers (20.0%) agreed with the statement, “I find it difficult to discuss my patients' use of tobacco with them,” χ^2^ (1, *N* = 420) = 6.87, *p* = 0.0087, this difference was not significant after Bonferroni correction. No other attitudes significantly differed between agencies.

**Figure 1:**
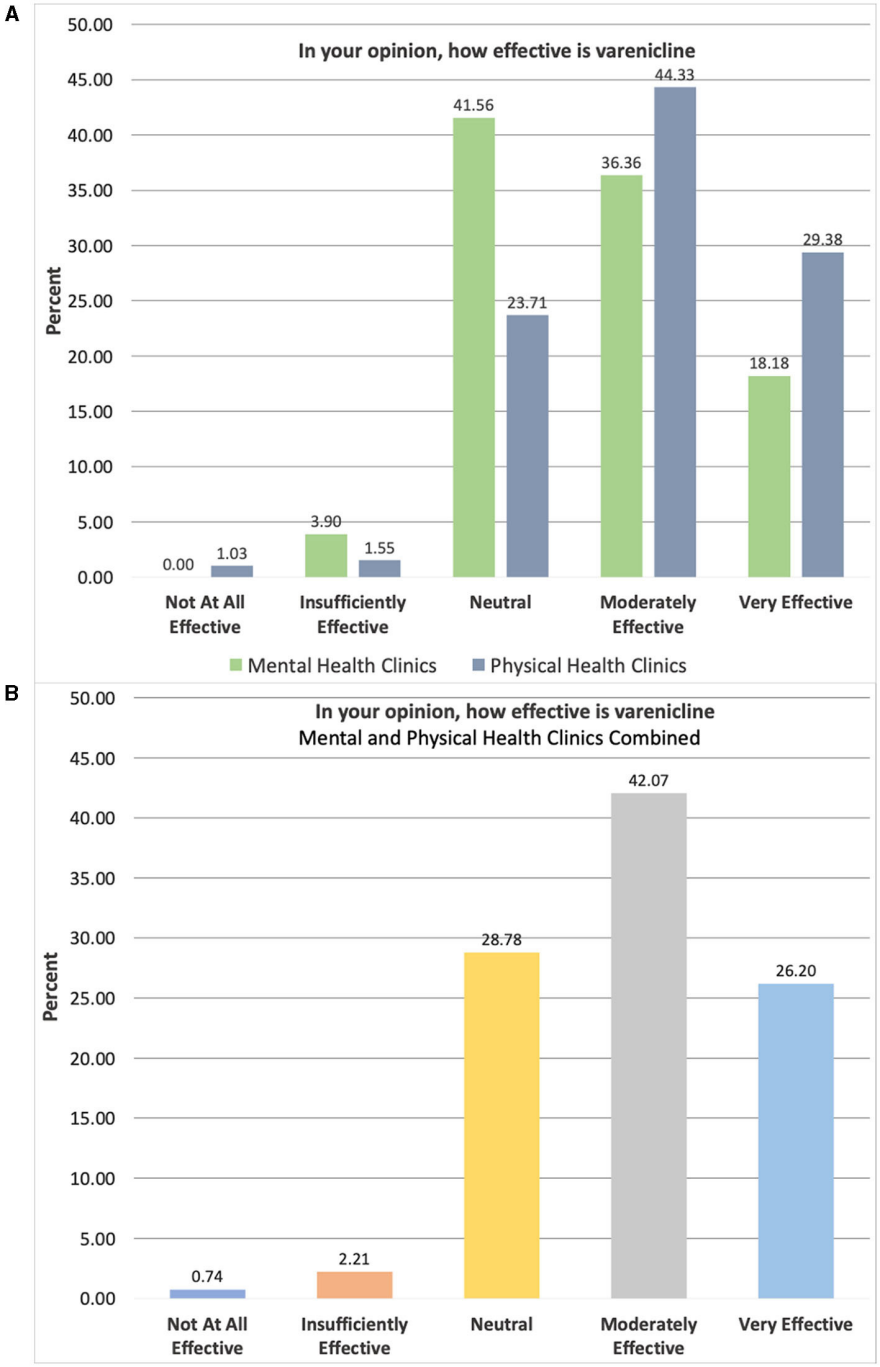
Breakdown of provider belief responses by clinic for varenicline efficacy. Items rated on 5-point Likert scale ranging from “Not At All Effective” (1) to “Very Effective” (5); **(A)** Physical Health Clinics = Department of Health Services: *n* = 194, Mental Health Clinics = Department of Mental Health: *n* = 77; **(B)** Clinics combined *N* = 271.

Both groups of providers expressed the belief that smoking cessation aids were “moderately effective” with median scores of 4 points on a 5-point Likert scale for all smoking aids listed (e.g., varenicline, nicotine path, and nicotine gum). The overall proportion of providers agreeing that smoking cessation aids were effective ranged from 53 to 68%. Agency providers significantly differed on one smoking cessation belief, such that DMH providers less strongly agreed that varenicline is effective for smoking cessation than DHS providers (see Figure 1), although median responses (4 points) were consistent between agencies, W = 9082.5, *p* = 0.003; Hodges-Lehmann difference estimate 0.000019 [95% CI: 0.000016, 1.00]. Dichotomized Chi-square results for this item on varenicline efficacy similarly showed a significant difference between agencies, such that a significantly greater proportion of DHS providers (73.7%) vs. DMH providers (54.6%) believed varenicline to be effective, χ^2^ (1, *N* = 271) = 9.35, *p* = 0.0022.

**Figure 2:**
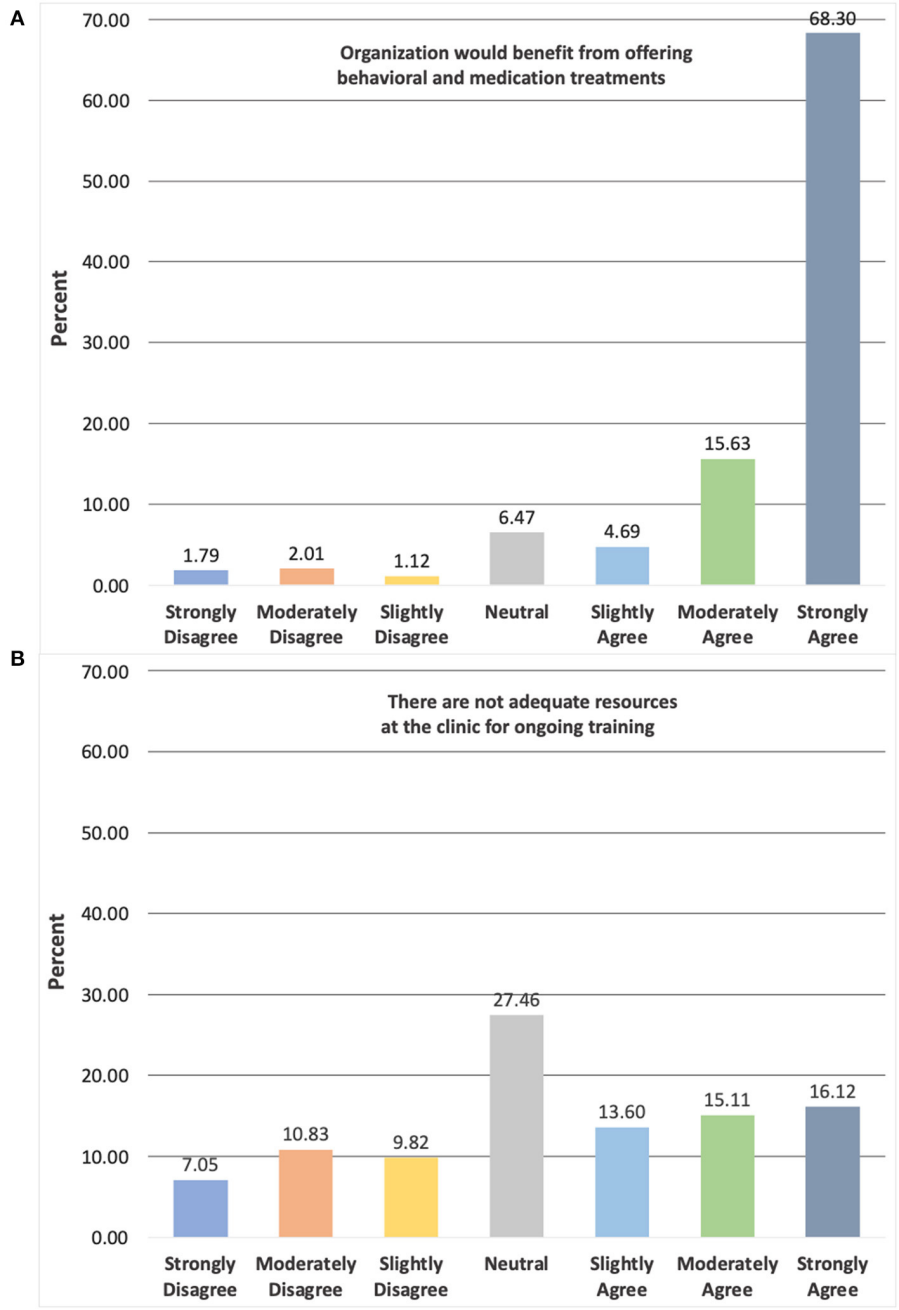
Breakdown of provider agreement for selected clinic-level items across agencies. Items rated on 7-point Likert scale ranging from “Strongly Disagree” (1) to “Strongly Agree” (7); total number of respondents differs for each survey item depending on response rates: **(A)** = 448, **(B)** = 397.

A detailed breakdown of providers' agreement ratings across the two agencies for select items focused on clinic-level factors is provided in Figure 2. Namely, items presented assess provider agreement on the organization benefiting from offering behavioral and medication treatments for smoking cessation as well as their clinic having inadequate resources for ongoing training in smoking cessation. Results show that the majority of providers “strongly agree” (68.3%) that the organization would benefit from offering both types of treatment, but providers reported more mixed attitudes regarding the adequacy of currently available clinic resources for ongoing training. Importantly, respondents (*n* = 367) also reported on whether they would be interested in receiving additional training to address tobacco use among their patients (yes vs. no). Interest was high across both DHS and DMH agencies with 76.3% of providers selecting “yes.”

## Discussion

Overall, this study sought to characterize and compare mental and physical health care provider attitudes about smoking cessation treatment within the Los Angeles County Health Agency. The safety net clinics included in this study provide care to low-income individuals with public health insurance, who are among those at greatest risk for developing TUD and experiencing negative health outcomes of cigarette smoking (4, 6). This is the first step (i.e., baseline assessment) in a large-scale implementation study designed to test specialty clinic-level intervention to boost tobacco cessation services and improve cessation rates. Our findings examining DHS vs. DMH clinic differences were consistent whether looking at dichotomized proportions of agreement vs. disagreement or graduated variations in agreement on the Likert scales. Across the two agencies, providers similarly and consistently reported that they believed smoking cessation aids to be moderately effective. Our hypothesis that DHS providers would report greater efficacy beliefs was supported for only one cessation aid, such that DMH providers less strongly agreed that varenicline is effective for smoking cessation. Varenicline, which is a nicotine antagonist approved as a first-line medication by the FDA, is evidenced to be an effective pharmacological treatment for smoking cessation and has shown superiority over nicotine replacement therapy and bupropion (8). DMH providers may have less strongly agreed that varenicline is effective for smoking cessation because of previously raised concern from case reports stating that varenicline may have exacerbated psychiatric symptoms, including suicidal behavior (8, 22); yet evidence demonstrates that varenicline does not increase neuropsychiatric adverse events (23). Overall, 63% of providers agreed that part of their job role is to treat or support the treatment of patients who smoke via pharmacotherapy. In line with our hypothesis, more than 75% of providers expressed interest in additional tobacco use training and moderately or strongly agreed that their organization would benefit from offering behavioral and medication treatments. Our findings also show that only 22% of providers agreed that medications for TUD can be effective without behavioral health counseling, suggesting that providers support the efficacy of combined pharmacotherapy and behavioral treatment, in line with best practices (24). Previous research suggests that training on smoking cessation treatment is not offered routinely to mental health professionals and thus may be an important barrier to address in DMH clinics (12). Similarly, 45% of providers from the current sample slightly to strongly agreed that there were not adequate resources at their clinic for ongoing TUD training, whereas a smaller proportion disagreed. Overall, these positive views toward smoking cessation treatment and suggested inadequacies in TUD training resources, show promise for directing resources to the implementation of specialized tobacco cessation services that combine medication and behavioral treatment in municipal health care agencies with the hopes of improving smoking cessation rates among high-risk and vulnerable populations (11, 14, 25).

Additionally, median responses show that providers only slightly or moderately agreed that they were prepared to identify and diagnose tobacco use among their patients. Consistent with our hypothesis and previous literature on TUD in patients with mental illness (12), DMH providers stated that identification of tobacco use was less in line with their job responsibilities. Yet, attitudes across other items were very similar for mental and physical health providers. Given the higher occurrence of smoking among people with mental illness and low SES (6), along with the negative impact smoking can have on psychotropic medication efficacy (26), community mental health clinics may be an especially important setting for addressing TUD symptoms. These results, consistent with previous work in the addictions field, suggest that Los Angeles County providers as well as safety net providers in general, may benefit from additional training on how to approach diagnosis, discussions, and treatment for TUD (12, 27, 28). Research suggests that professional health care trainings on smoking cessation interventions prove beneficial and improve rates of professional counseling along with point prevalence of smoking among their patients (13, 15). A follow-up survey by our group will be conducted after the conclusion of the county's implementation science project- consisting of trainings geared toward educating providers on smoking cessation medications- in order to assess provider attitudes following training.

The results of the present study should be considered in light of its strengths and limitations. Strengths include a unique insight into the differences between attitudes of physical and mental health care providers in a real-world setting. In addition, we obtained a substantial number of survey responses from providers with a variety of job roles, including medical providers, nurses, non-medical clinicians, and community health workers, who work within a large public health agency serving diverse community members. Limitations include the use of survey methodology, which is susceptible to response bias and respondent error, along with limited coded qualitative interviews or reports, which could bolster survey responses with more comprehensive understanding of provider perspectives and barriers to care, not captured via the Likert scales. It is possible that providers who self-selected to complete the survey were among those generally more enthusiastic about the treatment of TUD, which could present an overestimate of provider interest in and opinions toward TUD treatment (e.g., response rate of 49% and 70% from DMH and DHS clinic providers, respectively). While broad Los Angeles County metrics are available, a detailed characterization of provider respondents and their patients, such as demographic information, caseloads, and rates of tobacco use, were not available. Further, it is unclear to what extent modest differences in attitudes between DHS and DMH clinics translates into meaningful TUD counseling and treatment behavior. Lastly, the study sample was comprised of only Los Angeles County Health Agency providers and while results may be indicative of provider attitudes from similar safety net systems, findings may not generalize to smaller communities with dissimilar makeup or to private medical settings. For instance, providers in this study primarily treat patients with low incomes. Future research on this topic should be comprised of multisite data collection and include municipal health systems from various regions, states, and counties as well as rich qualitative data.

Overall, research suggests that population-based interventions are critical to reduce the health and economic burden of smoking-related diseases among U.S. adults (25), particularly among subpopulations with the highest prevalence of smokers, such as low-income individuals and those with mental illness (7). Providers from DHS and DMH clinics largely reported similar attitudes on smoking cessation treatment and expressed positive beliefs regarding the efficacy of smoking cessation aids. Our findings on mental and physical health care provider attitudes on smoking cessation treatment supports the implementation of comprehensive tobacco cessation treatment programs for safety net systems, which has the potential to yield large-scale public health benefits. More broadly, mental and physical safety net providers are essential in the health care system, as they deliver care to vulnerable populations who are disproportionately impacted by tobacco use. Patient interactions with safety net providers may serve as one of the few opportunities for these individuals to address their tobacco use (15). Equipping providers within the safety net systems with in-depth training on the identification and treatment of tobacco use, particularly in community mental health clinics, is vital in promoting tobacco cessation, especially among high-risk and vulnerable populations.

## Data Availability Statement

The raw data supporting the conclusions of this article will be made available by the authors, without undue reservation.

## Ethics Statement

The studies involving human participants were reviewed and approved by Los Angeles County Department of Public Health Institutional Review Board (IRB) #2018-11-776 and Los Angeles County Department of Mental Health Human Subjects Research Committee #339. The patients/participants provided their written informed consent to participate in this study.

## Author Contributions

TF and BH acquired funding and contributed to conception and design of the study. LM performed the statistical analysis. LM, LAR, and W-AB wrote the first draft of the manuscript. LR assisted with data collection and database organization. TF, BH, TD, NM, LR, BL, and SH reviewed and edited sections of the manuscript. All authors contributed to manuscript revision, read, and approved the submitted version.

## Funding

This work was supported by the California Tobacco-Related Disease Research Program [Grant 28CP-0040] awarded to TF and BH.

## Conflict of Interest

The authors declare that the research was conducted in the absence of any commercial or financial relationships that could be construed as a potential conflict of interest.

## Publisher's Note

All claims expressed in this article are solely those of the authors and do not necessarily represent those of their affiliated organizations, or those of the publisher, the editors and the reviewers. Any product that may be evaluated in this article, or claim that may be made by its manufacturer, is not guaranteed or endorsed by the publisher.
